# Cardiometabolic Risk Among Internal Medicine Specialists in Indonesia (CARMEINA): A Nationwide Study

**DOI:** 10.1016/j.jacasi.2025.09.024

**Published:** 2025-11-26

**Authors:** Eka Ginanjar, Simon Salim, Sally Aman Nasution, Lugyanti Sukrisman, Evy Yunihastuti, Andhika Rachman, Ceva Wicaksono Pitoyo, Hayatun Nufus, Muhadi Muhadi, Hermawan Susanto, Siprianus Ugroseno Yudho Bintoro, Darmadi Darmadi, Asri Ludin Tambunan, Hery Djagat Purnomo, Andi Makbul Aman, Anggoro Budi Hartopo, Mohammad Robikhul Ikhsan, Selfie Selfie, Ahmad Mekah, Ervita Ritonga, Indra Wijaya, Gede Wira Mahadita, Wira Gotera, Radiyati Umi Partan, Zen Ahmad, Azzaki Abubakar, Muhammad Diah, Nur Samsu, Wachid Putranto, Tatar Sumandjar, Reinaldo Alexander, Johana Prihatini, Dani Rosdiana, Juwanto Wakimin, Cecilia Hendratta, Linda Wilhelma Ancella Rotty, Akmal Mufriady Hanif, Harnavi Harun, Astried Indrasari, Kuntjoro Yakti, Erwanto Budi Winulyo, Erwin Erwin, Lukman Pura, Djallalluddin Djallalluddin, Abimanyu Abimanyu, Giri Satriya, Sutiadi Kusuma, Andi M.H. Panjaitan, Suharno Hakim, Elfiani Elfiani, Budiman Gunawan, Annisa Puspitasari Nachrowi, Kongko H. Nursetiyanto, Kurniakin W.S. Girsang, Arif Koswandi, Erwin A. Pangkahila, Andreas N.F. Lewai, Joko Anggoro, Dayang Nurbayati, Fahmi R. Darkuthni, Arfan Sanusi, Petrus Irianto, Komariatun Komariatun, Annelin Kurniati, Haeril Aswar, Nelyan H. Mokoginta, Leily D. Pawa, Edwin Ambar, Riswan Riswan, Feliks Duwit

**Affiliations:** a Faculty of Medicine Universitas Indonesia, Cipto Mangunkusumo Hospital, Jakarta, Special Capital Region of Jakarta, Jakarta, Indonesia; b Faculty of Medicine Universitas Indonesia, Persahabatan Central General Hospital, Jakarta, Special Capital Region of Jakarta, Jakarta, Indonesia; c Faculty of Medicine Universitas Airlangga, Dr Soetomo General Academic Hospital, Surabaya, East Java; d Faculty of Medicine Universitas Sumatera Utara, Medan, North Sumatra; e Faculty of Medicine Universitas Diponegoro, Dr Kariadi Hospital, Semarang, Central Java; f Universitas Hasanuddin Hospital, Makassar, South Sulawesi; g Faculty of Medicine, Public Health, and Nursing Universitas Gadjah Mada, Dr Sardjito Hospital, Yogyakarta, Special Region of Yogyakarta; h Balaraja Hospital, Tangerang, Banten; i Sari Asih Sangiang Hospital, Tangerang, Banten; j Faculty of Medicine Universitas Padjadjaran, Hasan Sadikin Hospital, Bandung, West Java; k Faculty of Medicine Universitas Udayana, Prof dr I Goesti Ngoerah Gde Ngoerah General Hospital, Denpasar, Bali; l Faculty of Medicine Universitas Sriwijaya, Mohammad Hoesin General Hospital, Palembang, South Sumatra; m School of Medicine Universitas Syiah Kuala, Dr Zainoel Abidin Hospital, Banda Aceh, Aceh; n Faculty of Medicine Universitas Brawijaya, Saiful Anwar General Hospital, Malang, East Java; o Faculty of Medicine Universitas Sebelas Maret, Dr. Moewardi General Hospital, Surakarta, Central Java; p Siloam Hospitals Bekasi Sepanjang Jaya, Bekasi, West Java; q Primaya Hospital, Bekasi, West Java; r Faculty of Medicine Universitas Riau, Arifin Achmad General Hospital, Pekanbaru, Riau; s Faculty of Medicine Universitas Sam Ratulangi, Prof dr R. D. Kandou Hospital, Manado, North Sulawesi; t Faculty of Medicine Universitas Andalas, Dr M. Djamil General Hospital, Padang, West Sumatra; u Abdul Wahab Sjahranie Hospital, Samarinda, East Kalimantan; v Hermina Hospital, Bogor, West Java; w Indonesia Red Cross General Hospital, Bogor, West Java; x Faculty of Medicine Lampung University, Abdul Moelok Hospital, Bandar Lampung, Lampung; y Faculty of Medicine Universitas Lambung Mangkurat, Ulin Hospital, Banjarmasin, South Kalimantan; z Juanda Hospital, Kuningan, West Java; aa Gunung Jati General Hospital, Cirebon, West Java; bb Emanuel Hospital, Banjarnegara, Central Java; cc Faculty of Medicine Universitas Jenderal Soedirman, Dr Margono Soekarjo Hospital, Purwokerto, Central Java; dd Faculty of Medicine Universitas Jambi, Raden Mattaher Hospital, Jambi, Jambi; ee Kharitas Bhakti Hospital, Pontianak, West Kalimantan; ff Mayapada Hospital Jakarta Selatan, Jakarta, Special Capital Region of Jakarta; gg Engku Haji Daud Hospital, Bintan, Riau Islands; hh Awal Bros Hospital Batam, Batam, Riau Islands; ii Karitas Weetabula Hospital, West Sumba, East Nusa Tenggara; jj Faculty of Medicine Universitas Nusa Cendana, Prof Dr W. Z. Johannes Kupang Regional General Hospital, Kupang, East Nusa Tenggara; kk Faculty of Medicine University of Mataram, West Nusa Tenggara Regional General Hospital, Mataram, West Nusa Tenggara; ll dr Doris Sylvanus Hospital, Palangkaraya, Central Kalimantan; mm Faculty of Medicine Universitas Tadulako, Palu, Central Sulawesi; nn Faculty of Medicine Alkhairaat University, Palu, Central Sulawesi; oo Jayapura Regional General Hospital, Jayapura, Papua; pp Faculty of Medicine Universitas Bangka Belitung, Pangkal Pinang, Bangka Belitung Islands; qq Harapan dan Doa Regional Hospital, Bengkulu, Bengkulu; rr Faculty of Medicine Universitas Hasanuddin, Makassar, South Sulawesi; ss Aloei Saboe Hospital, Gorontalo, Gorontalo; tt Piru Hospital, West Seram, Maluku; uu Faculty of Medicine Universitas Negeri Khairun, Chasan Boesoirie General Hospital, Ternate, North Maluku; vv Faculty of Medicine Universitas Papua, John Piet Wanane Regional General Hospital, Sorong, Southwest Papua; ww Faculty of Medicine Universitas Papua, Manokwari, West Papua, Indonesia

**Keywords:** ASCVD, cardiometabolic syndrome risk, internal medicine specialist SCORE-2 Asia Pacific

## Abstract

**Background:**

Cardiovascular and cerebrovascular diseases are the top global causes of mortality. Internal medicine professionals are vital in educating the public on prevention, yet they themselves may experience the very risk factors they warn against.

**Objectives:**

This study aimed to assess cardiometabolic risk factors among Indonesian internal medicine specialists to raise awareness among health care workers.

**Methods:**

This cross-sectional study was conducted by the Indonesian Society of Internal Medicine (INASIM). Our study recruited 1,568 internal medicine specialists from 39 INASIM branches in Indonesia using clustered random sampling (December 2023-March 2024). Cardiometabolic risk was calculated using atherosclerotic cardiovascular disease (ASCVD) risk scores and SCORE-2 (Systematic Coronary Risk Evaluation-2) Asia Pacific.

**Results:**

The mean age of 1,064 participants who completed the study was 45 ± 10.4 years. The prevalences of dyslipidemia, obesity, hypertension, and diabetes mellitus were 84.6% (95% CI: 82.4%-86.9%), 61.4% (95% CI: 58.5%-64.3%), 20.3% (95% CI: 17.9%-22.7%), and 10.2% (95% CI: 8.4%-12.1%), respectively. Participants’ 10-year ASCVD risk scores were 6.0% (95% CI: 4.4%-8.1%) high, 18.5% (95% CI: 15.7%-21.7%) intermediate, 9.6% (95% CI: 7.5%-12.1%) borderline, and 66.0% (95% CI: 62.2%-69.6%) low. Their SCORE-2 Asia Pacific risk scores were 1.2% (95% CI: 0.5%-2.5%) very very high, 10.7% (95% CI: 8.3%-13.7%) very high, 46.5% (95% CI: 42.1%-50.9%) high, and 41.6% (95% CI: 37.3%-46.1%) moderate.

**Conclusions:**

The prevalences of dyslipidemia and obesity were high among internal medicine specialists in Indonesia. The prevalences of highest risk category profile were 6.0% (ASCVD) and 1.2% (SCORE-2).

Cardio-cerebrovascular diseases are the leading cause of mortality worldwide.^1^^,^^2^ Since the advent of the Framingham heart study,^3^ studies like INTERHEART (Effect of potentially modifiable risk factors associated with myocardial infarction in 52 countries)^4^ have identified several risk factors for cardiovascular disease (CVD). Most of these factors have been incorporated in risk score tools like atherosclerotic cardiovascular disease (ASCVD) risk estimator and SCORE-2 (Systematic Coronary Risk Evaluation-2),^5^^,^^6^ which also include age, sex, smoking status, history of diabetes mellitus, hypertension therapy, blood pressure, and lipid profile. ASCVD risk estimator and SCORE-2 are considered as tools for estimation of risk in patients from many countries, including Indonesia.

Most CVD cases occurred in developing countries;^7^ the prevalence in Indonesia was estimated to be 0.8% in 2023.^8^ Health care professionals, including internal medicine physicians, play a crucial role in educating the general population about CVD prevention, yet they may be prone to the same risk factors. Internal medicine specialists play a significant role in identifying and managing cardiometabolic risk in populations. Thus, we aimed to evaluate cardiometabolic risk factors among internal medicine specialists in Indonesia to raise awareness among health care workers.

## Materials and Methods

### Design

Led by the Indonesian Society of Internal Medicine (INASIM/PAPDI), this nationwide study employed a cross-sectional design with clustered random sampling across INASIM branches (national representative), conducted between December 2023 and March 2024.

### Study participants

Using sample size formulation for simple random sampling study, with 95% CI, 5% margin of error, we found the required sample size is 1,199, which is equivalent to about 20% of the population, Subjects were randomly selected to include 20% of 5,436 internal medicine specialists from 39 branches (district) across Indonesia. In December 2023, personal invitations were issued to internal medicine specialists; if the internal medicine specialist refused to participate, re-randomization from the same branch was done until target sample was acquired in January 2024. All internal medicine specialists were eligible for inclusion. The only exclusion criterion was incomplete data.

### Data collection

Data collection occurred in 2 sequential phases; the maximum interval between phases was 7 days. In the first phase, participants completed self-administered online questionnaires, using Google Form, containing demographic characteristics, medical history, lifestyle (smoking history, sleep duration, and physical activity), and dietary habit (salt, coffee, and alcohol consumption). The questions were self-reported by the participants and included history of smoking, amount of salt consumption (g/d), alcohol (glass/d), coffee (glass/d), duration of sleep per night (h/d), duration of moderate intensity exercise (h/wk), duration of vigorous intensity exercise (h/wk). The second phase comprised standardized physical and laboratory examinations conducted at 39 branch sites. Before enrollment, all subjects provided electronically administered informed consent. Physical examinations included anthropometric measurements (eg, height, weight) and blood pressure assessments performed by trained medical staff at each site using standardized stadiometer, weight scales, and sphygmomanometer. Venous blood samples were collected after a 12-hour fasting period and analyzed by a single standardized nationwide laboratory to ensure consistency in biochemical profiling. For the reference value, we used guidelines from the National Consensus, including the *Konsensus Penegendalian Diabetes Melitus 2011* and *Konsensus Lipid*. HbA1C measurement was carried out using the high-performance liquid chromatography ion exchange method, and has been standardized by the National Glycohemoglobin Standardization Program with a reference value of prediabetes of 5.7% to 6.4% and diabetes >6.5%. The results of physical and laboratory examinations were collected by each site investigator via Google Form.

### Data analysis

Categorical data are presented in frequency (n) and percentage (%), and continuous data are presented as mean ± SD or median (IQR).

Cardiometabolic risk was calculated by the collaborators using ASCVD risk score^5^ (low, <5%; borderline, 5% to <7.5%; intermediate, 7.5% to <20%; and high, ≥20%) and SCORE-2 Asia Pacific risk score (low, <5%; moderate, 5% to <10%; high, 10% to <20%; very high, 20% to <30%; and very very high, ≥30%).^6^ ASCVD risk score^5^ were calculated using the American College of Cardiology/American Heart Association 2014 web-based ASCVD Risk Estimator tool. SCORE-2 Asia Pacific was calculated using SCORE2 Asia Pacific risk charts for the prediction of 10-year risk in 4 Asia-Pacific risk regions.^6^

Analysis of risk profiles and other participant characteristics was done using a cumulative logic model. Furthermore, we analyzed the agreement between the 10-year ASCVD and SCORE-2 Asia Pacific risk scores, in a harmonized category system (low, <5%; intermediate, 5% to <20%; and high, ≥20%), using Cohen's weighted kappa. The values obtained from this analysis were then interpreted based on definitions outlined by Altman:^9^ 10-year ASCVD risk estimates for those aged 40 to 79 and lifetime ASCVD risk estimates for those aged 20 to 59, both without history of ASCVD and SCORE-2 Asia Pacific estimates for those aged 40 to 69 without diabetes mellitus or prior ASCVD event.

### Ethical approval

This study was approved by the Health Research Ethics Committee - Faculty of Medicine Universitas Indonesia and Dr Cipto Mangunkusumo National Hospital (KET-1366/UN2.F1/ETIK/PPM.00.02/2023). Participation consent was obtained from all subjects before filling out the questionnaire.

## Results

**Figure 1:**
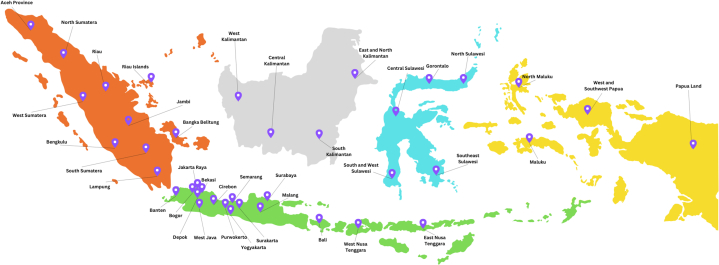
Map Distribution of Study Participants Across Indonesia This figure illustrates the branch distribution of internal medicine specialists who participated in the CARMEINA (Cardiometabolic Risk among Internal Medicine Specialists in Indonesia) study. Participant locations were geocoded based on self-reported Indonesian Society of Medicine branches and visualized in the mean of Indonesia map to depict the national spread. A total of 1,024 participants were included, representing all regions, with highest branch concentrations observed in Java Island. This spatial distribution highlights the diverse demographic representation and supports the generalizability of findings regarding cardiometabolic risk among internal medicine specialists in Indonesia.

A total of 1,568 internal medicine specialists were invited to participate, of whom 1,136 filled out the questionnaire. Seventy-two participants withdrew for various reasons, such as residing in remote areas (far from intended standardized laboratories), experiencing force majeure events (eg, floods), or having busy schedules. Consequently, 1,064 participants completed the study. The distribution of locations is shown in Figure 1. Demographic characteristics of participants are provided in Table 1. Most participants (56.4%) were between the ages of 35 and 49; 57.5% were men. Almost all participants practiced in urban regions (80.7%). Most participants’ working hours were ≥55 h/wk (52.4%).

**Table 1:** Demographic Characteristics of Study Participants

Age, y	
<35	139/1,064 (13.1)
35 to <50	600/1,064 (56.4)
50 to <65	264/1,064 (24.8)
≥65	61/1,064 (5.7)
Sex	
Male	612/1,064 (57.5)
Female	452/1,064 (42.5)
Practice location category	
Category based on Central Bureau of Statistics (Indonesia)	
Urban	665/1,064 (62.5)
Rural	203/1,064 (19.1)
Urban-rural	194/1,064 (18.2)
Average working hours, h/wk	
<55	506/1,064 (47.6)
≥55	558/1,064 (52.4)

Values are n/N (%).

We assessed lifestyle cardiometabolic syndrome risk factors, as shown in Table 2. Alcohol was consumed by 3.9% (41 of 1,064; 95% CI: 2.7%-5.0%) of participants; 55.0% (586 of 1,064; 95% CI: 52.1-58.1%) regularly consumed coffee; 11.2% (119 of 1,064; 95% CI: 9.4%-13.2%) had a history of smoking; and 25.4% (270 of 1,064; 95% CI: 22.7%-28.1%) of participants consumed salt >5 g/d. Most participants did not meet the recommended sleep duration (63%) (670 of 1,064; 95% CI: 60.1%-66.0%). Only a small portion of participants performed the recommended aerobic activity durations, with 17.0% (181 of 1,064; 95% CI: 14.8%-19.3%) engaged in moderate intensity and 11.0% (117 of 1,064; 95% CI: 9.1%-12.9%) engaged in high-intensity activity.

**Table 2:** Lifestyle and Dietary Habits

Alcohol consumption	
None	1023/1,064 (96.1)
<1 glass/d	35/1,064 (3.3)
≥1 glass/d	6/1,064 (0.6)
Daily coffee consumption	
None	478/1,064 (44.9)
1 glass/d	344/1,064 (32.3)
≥2 glass/d	242/1,064 (22.7)
Cigarette smoking	
Not smoking	945/1,064 (88.8)
Active smoking	30/1,064 (2.8)
Stopped smoking	89/1,064 (8.4)
Salt consumption >5 g/d	270/1,064 (25.4)
Sleep duration <7 h/d	670/1,064 (63.0)
Moderate intensity aerobic exercise <150 min/wk	883/1,064 (83.0)
High-intensity aerobic exercise <75 min/wk	947/1,064 (89.0)

Values are n/N (%).

Anthropometric measurements, blood pressure, and selected biochemical measurements are provided in Table 3. The prevalence of obesity was 61.4% (653 of 1,064; 95% CI: 58.5%-64.3%), with 71.4% (760 of 1,064; 95% CI: 68.6-74.1) of participants having central obesity. Blood pressure measurements revealed that 20.3% (216 of 1,064; 95% CI: 17.9%-22.7%) of participants had hypertension. The prevalence of diabetes mellitus based on examinations was 10.2% (108 of 1,064; 95% CI: 8.4%-12.1%). Lipid profiles revealed that 56.6% (602 of 1,064; 95% CI: 53.6%-59.6%) of participants had total cholesterol ≥200 mg/dL, 84.6% (900 of 1,064; 95% CI: 82.4%-86.9%) had low-density lipoprotein cholesterol ≥100 mg/dL, 15.5% (165 of 1,064; 95% CI: 13.4%-17.7%) had high-density lipoprotein cholesterol <40 mg/dL, and 24.0% (255 of 1,064; 95% CI: 21.3%-26.7%) had triglycerides ≥150 mg/dL. Most participants (68.7%) (731 of 1,064; 95% CI: 65.9%-71.5%) had estimated glomerular filtration rate ≥90 mL/min/1.73 m^2^.

**Table 3:** Anthropometric Measurements, Blood Pressure, and Selected Biochemical Measurements

BMI, kg/m^2^	
<18.5	13/1,064 (1.2)
18.5-<23	184/1,064 (17.3)
23-<25	214/1,064 (20.1)
25-<30	440/1,064 (41.4)
≥30	213/1,064 (20.0)
Abdominal circumference	
<90 cm (male) or <80 cm (female)	304/1,064 (28.6)
≥90 cm (male) or ≥80 cm (female)	760/1,064 (71.4)
Blood pressure	
SBP <130 mm Hg and DBP <85 mm Hg	690/1,064 (64.8)
Pre-hypertension (SBP 130-139 mm Hg and/or DBP 85-89 mm Hg)	158/1,064 (14.8)
Hypertension Grade 1 (SBP 140-159 mm Hg and/or DBP 90-99 mm Hg)	162/1,064 (15.2)
Hypertension Grade 2 (SBP 160-179 mm Hg and/or DBP 100-109 mm Hg)	54/1,064 (5.1)
FBG, mg/dL	
<100	757/1,064 (71.1)
100–125	244/1,064 (22.9)
>125	63/1,064 (5.9)
HbA1c (glycosylated hemoglobin)	
HbA1c <5.7%	739/1,064 (69.5)
HbA1c 5.7%-<6.5%	240/1,064 (22.6)
HbA1c ≥6.5%	85/1,064 (8.0)
FBG and HbA1c	
FBG <100 mg/dL and HbA1c <5.7%	598/1,064 (56.2)
FBG 100–125 mg/dL or HbA1c 5.7%-<6.5%	358/1,064 (33.6)
FBG >125 mg/dL or HbA1c ≥6.5%	108/1,064 (10.2)
Total cholesterol, mg/dL	
<200	462/1,064 (43.4)
≥200	602/1,064 (56.6)
LDL-C, mg/dL	
<100	164/1,064 (15.4)
≥100	900/1,064 (84.6)
HDL-C, mg/dL	
<40	165/1,064 (15.5)
≥40	899/1,064 (84.5)
Triglyceride, mg/dL	
<150	809/1,064 (76.0)
≥150–199	255/1,064 (24.0)
eGFR (CKD-EPI), mL/min/1.73 m^2^	
<15	1/1,064 (0.1)
15-<30	3/1,064 (0.3)
30-<45	6/1,064 (0.6)
45-<60	29/1,064 (2.7)
60-<90	294/1,064 (27.6)
≥90	731/1,064 (68.7)

Values are n/N (%).

BMI = body mass index; CKD-EPI = chronic kidney disease epidemiology collaboration; DBP = diastolic blood pressure; eGFR = estimated glomerular filtration rate; FBG = fasting blood glucose; HbA1c = glycated hemoglobin; HDL-C = high-density lipoprotein cholesterol; LDL-C = low-density lipoprotein cholesterol; SBP = systolic blood pressure.

The 10-year ASCVD risk is shown in Table 4 from 638 participants who met the 10-year ASCVD risk calculation criteria; most had a risk of <5% (421 of 638; 66.0%, 95% CI: 62.2%-69.6%). However, the lifetime ASCVD risk was >39% in most of the 957 participants whose data met the calculation range criteria (696 of 957; 72.7%, 95% CI: 69.8%-75.5%). The 10-year and lifetime ASCVD risk profiles based on INASIM branch and sex are shown in Supplemental Figures 1 and 2.

**Table 4:** ASCVD Risk in 10 Years and Lifetime

	10-Year ASCVD Risk				Lifetime ASCVD Risk	
	<5%	5%-<7.5%	7.5%-<20%	≥20%	<39%	≥39%
Age, y						
25-35	0/638 (0.0)	0/638 (0.0)	0/638 (0.0)	0/638 (0.0)	52/957 (5.4)	87/957 (9.1)
35-<50	307/638 (48.1)	18/638 (2.8)	10/638 (1.6)	0/638 (0.0)	178/957 (18.6)	422/957 (44.1)
50-<65	113/638 (17.7)	42/638 (6.6)	91/638 (14.3)	7/638 (1.1)	31/957 (3.2)	187/957 (19.54)
≥65	1/638 (0.2)	1/638 (0.2)	17/638 (2.7)	31/638 (4.9)	0/957 (0.0)	0/957 (0.0)
Sex						
Male	117/638 (27.7)	53/638 (8.3)	113/638 (17.7)	36/638 (5.6)	95/957 (9.9)	426/957 (44.5)
Female	244/638 (38.2)	8/638 (1.3)	5/638 (0.8)	2/638 (0.3)	166/957 (17.4)	270/957 (28.21)
Practice location category						
Urban	269/638 (42.2)	36/638 (5.6)	93/638 (14.6)	25/638 (3.9)	171/957 (17.9)	411/957 (43.0)
Rural	66/638 (10.3)	17/638 (2.7)	13/638 (2.0)	12/638 (1.9)	48/957 (5.0)	146/957 (14.35)
Urban-rural	86/638 (13.5)	8/638 (1.3)	12/638 (1.9)	1/638 (0.2)	42/957 (4.4)	137/957 (15.3)
Total	421/638 (66.0)	61/638 (9.6)	118/638 (18.5)	38/638 (6.0)	261/957 (27.3)	696/957 (72.7)

Values are n/N (%).

ASCVD = atherosclerotic cardiovascular disease.

The SCORE-2 risk score is shown in Table 5. Of 514 participants who met the SCORE-2 Asia Pacific (nondiabetes) risk calculation range, most had a risk of 10% to 20% (239 of 514; 46.5%, 95% CI: 42.1%-50.9%). In the extended application, we compared SCORE-2 Asia Pacific risk between nondiabetic and diabetic groups. As we expected, the diabetic group had a higher risk compared with the nondiabetic group, as diabetes mellitus is considered as one of the CVD risks. Supplemental Figures 3 and 4 show the SCORE-2 Asia Pacific risk profile based on INASIM branch location and sex in nondiabetic and diabetic participants.

**Table 5:** SCORE-2 Asia Pacific Risk Score in Nondiabetic and Diabetic Participants

	SCORE-2 Asia Pacific (Nondiabetes) Risk Score				SCORE-2 Asia Pacific (Diabetes) Risk Score			
	<10%	10%-<20%	20%-<30%	≥30%	<10%	10%-<20%	20%-<30%	≥30%
Age, y								
40-<50	206/514 (40.1)	70/514 (13.6)	2/514 (0.4)	0/514 (0.0)	13/94 (13.8)	14/94 (14.9)	0/94 (0.0)	0/94 (0.0)
50-<65	8/514 (1.6)	169/514 (32.9)	43/514 (8.4)	1/514 (0.2)	1/94 (1.1)	44/94 (46.8)	14/94 (14.9)	0/94 (0.0)
65-69	0/514 (0.0)	0/514 (0.0)	10/514 (2.0)	5/514 (1.0)	0/94 (0.0)	0/94 (0.0)	6/94 (6.4)	2/94 (2.1)
Sex								
Male	94/514 (18.3)	132/514 (25.7)	43/514 (8.4)	6/514 (1.17)	6/94 (6.4)	45/94 (47.9)	17/94 (18.1)	2/94 (2.1)
Female	120/514 (23.35)	107/514 (20.8)	12/514 (2.3)	0/514 (0.0)	8/94 (8.6)	13/94 (13.8)	3/94 (3.2)	0/94 (0.0)
Practice location category								
Urban	139/514 (27.0)	156/514 (30.4)	38/514 (7.4)	3/514 (0.6)	6/94 (6.4)	40/94 (42.6)	15/94 (16.0)	1/94 (1.1)
Rural	28/514 (5.5)	42/514 (8.2)	7/514 (1.4)	3/514 (0.6)	5/94 (5.3)	12/94 (12.8)	4/94 (4.3)	1/94 (1.1)
Urban-rural	47/514 (9.1)	41/514 (8.0)	10/514 (2.0)	0/514 (0.0)	3/94 (3.2)	6/94 (6.4)	1/94 (1.1)	0/94 (0.0)
Total	214/514 (41.6)	239/514 (46.5)	55/514 (10.7)	6/514 (1.2)	14/94 (14.9)	58/94 (61.7)	20/94 (21.3)	2/94 (2.1)

Values are n/N (%).

Cohen's weighted kappa test was performed on 506 participants. The Cohen’s Weighted Kappa value of the 10-year ASCVD and SCORE-2 results was 0.212 (95% CI: 0.178-0.246). According to Altman,^9^ this value indicates a “fair” level of agreement between the 2 raters (10-year ASCVD and SCORE-2).

## Discussion

Cardiometabolic syndrome is defined as a group of metabolic disorders that can lead to CVDs. These disorders include central obesity, dyslipidemia, and type 2 diabetes mellitus. Sociodemographic factors, health behaviors, and neuropsychiatric outcomes significantly contribute to the development of cardiometabolic syndrome.^10^ In this study we expected external factors of cardiometabolic issues among internal medicine specialists to differ from the general population, including psychosocial factors (work stress), physical activity, sleep duration, education level, and dietary patterns. Our physician subjects had excessive workloads, as most worked ≥55 h/wk (52.4%). According to the World Health Organization (WHO), exposure to long working hours (>55 h/wk) was associated with higher risk of stroke (pooled risk ratio 1.35) and ischemic heart disease (pooled risk ratio 1.17).^11^, ^12^, ^13^ Wu et al^14^ reported that 61.1% of 20,786 doctors in China slept less than 7 hours per day, similar to our findings. Moreover, only 21.4% of our participants engaged in regular aerobic activity recommended by WHO.^15^ In addition, 25.4% of participants had high sodium consumption, but it was lower than that of the general Indonesian population (52.7%), based on the 2018 Indonesia Basic Health Research Survey.^16^

We found a remarkably low proportion of physicians who smoked cigarettes (2.8% active and 8.4% former smokers), which was lower than that of the Indonesian general population (33.5%),^17^ and lower than the 21% smoking prevalence among physicians shown in a meta-analysis by Besson et al,^18^ which primarily included studies from European countries. This positive finding was one of a few variables indicating that internal medicine specialists in Indonesia not only have the knowledge, but also better behavior than that of the general population with regard to smoking.

A meta-analysis study on problematic alcohol consumption among 51,680 physicians predominantly from European countries found an increase from 16.3% in 2006-2010 to 26.8% in 2017-2020, which was higher than our study (3.9%).^19^ One of the reasons for low alcohol consumption habits in our study is different socio-cultural conditions between Indonesia and other countries. According to the Indonesian Health Survey 2023, alcohol consumption is low in Indonesia's general population (2.2%), which was in line with our findings.^8^

The prevalence of hypertension among participants was 20.3%, which was lower than the 30.8% reported in the general population of Indonesia.^8^ Similar results were observed by Kurtul et al,^20^ who found that the prevalence of hypertension among physicians was also lower than in the general population. Likewise, the prevalence of diabetes mellitus among our participants was 10.2%, lower than the 11.7% reported for the general Indonesian population.^8^ Another study reported a prevalence of type 2 diabetes mellitus among primary health care physicians at 11.1%. This difference may have been due to our larger sample size (1,064 vs 495, respectively).^21^ Our study design does not help explain the phenomenon, thus, further study is needed to elucidate factors that help lower the prevalence of hypertension and diabetes in internal medicine specialists.

Most of our participants were classified as obese, both based on body mass index (BMI ≥25) and abdominal circumference measurements (≥90 cm for men and ≥80 cm for women). Similarly, Nair et al^22^ reported that most physicians were also categorized as obese according to BMI in a South Indian study. The prevalence of obesity in our study was higher compared with the general population in Indonesia. In addition, the prevalence of low-density lipoprotein >100 mg/dL among participants was 84.6%, which was significantly higher than the 63.5% reported in the general Indonesian population.^8^ Similar trends were also observed among physicians in a study by Ramachandran et al in India.^23^

Our study revealed that 72.7% of internal medicine specialists have a high lifetime risk of ASCVD (≥39%). Most internal medicine specialists’ 10-year ASCVD risk was <5% (66.0%). A similar finding was in a study conducted in East Java, Indonesia, that most participants (65.6%) had 10-year risk of CVD <10% using WHO/International Society of Hypertension risk charts calibrated for use in Southeast Asia regions B (SEAR B).^24^ Internal medicine specialists’ 10-year ASCVD risk was ≥7.5% in 23.5% of our participants. This high risk of ASCVD was in agreement with the prevalences of hypertension, diabetes mellitus, and dyslipidaemia, which were quite high. Similar ASCVD risk results were reported among physicians in Chittagong, Bangladesh.^25^ An et al^26^ reported that some adults may have a low 10-year risk, but a high predicted lifetime ASCVD risk. A similar phenomenon was observed in our study. Young adults have a low 10-year ASCVD predicted risk, but a high lifetime risk, leading to higher incidence of subclinical ASCVD. Targeting this subgroup for preventive measures is crucial.^26^

Using SCORE-2 Asia Pacific, most internal medicine specialists (58.4%) had high to very very high risk of CVD. Similarly, a SCORE2 study in a Portuguese population, noted that the majority had a high risk of CVD.^27^ SCORE2 is designed to calculate the risk of CVD for individuals without prior CVD or diabetes mellitus. The rationale is that diabetic individuals are generally considered to be at high risk of CVD.^6^ This aligns with our findings, as the diabetes group had a higher risk of CVD than the nondiabetes group, estimated by SCORE-2 Asia Pacific.

In our study, 58.4% of internal medicine specialists had a CVD risk of ≥10% in 10 years using the SCORE-2 Asia Pacific. This proportion was higher compared with the 10-year ASCVD, in which 23.5% internal medicine specialists had an ASCVD risk of ≥7.5%. We noted a fair level of agreement between 10-year ASCVD and SCORE-2 Asia Pacific risk scores. All the 10-year ASCVD high risk category internal medicine specialists were also in the SCORE-2 Asia Pacific high-risk category. However, most of the 10-year ASCVD low-risk category (<5% risk) were in the SCORE-2 Asia Pacific intermediate-risk category, indicating a 5% to 19.9% chance that an individual will experience a major cardiovascular event in the next 10 years, according to SCORE-2 Asia Pacific. The SCORE2 tool appears to categorize patients as having a higher risk compared with the 10-year ASCVD risk scoring system. A retrospective study of people living with HIV had similar findings, with a larger proportion of participants having higher risk of CVD according to the SCORE-2 tool compared with the 10-year ASCVD scoring system.^28^ There are several possible reasons for this difference. SCORE2 is intended to estimate CVD risk in individuals without diabetes, whereas the ASCVD tool takes diabetes into consideration. In addition, the SCORE2 Asia Pacific tool is the first model available that has been recalibrated to several regions grouped according to age-standardized CVD mortality rates. Meanwhile, the 10-year ASCVD cohorts included African American or White participants.^5^^,^^6^ SCORE2 broadens the risk assessment to encompass population-level risks from a global viewpoint, beyond the individual patient. An individual living in a "very high-risk country" (CVD mortality rates ≥300 CVD deaths per 100,000 population), including Indonesia, may have increased cardiovascular risk despite the lack of traditional risk factors; thus, other internal or external factors specific to their country may account for this higher risk. Despite variations in the guidelines regarding the risk stratification system, the underlying principles remain similar. Both use risk prediction models as a foundation for a comprehensive strategy to optimize cardiovascular health.^29^

The CVD risk comparison charts show that female internal medicine specialists tended to have lower risk than male internal medicine specialists, as assessed using the 10-year ASCVD and SCORE-2 Asia Pacific. Women not only by nature had lower risk of developing CVD,^30^ but they also had a lower cardiometabolic risk profile as measured by ASCVD and SCORE-2 Asia Pacific in our study.

This study represents the first comprehensive assessment of cardiometabolic risk profiles among health care workers, particularly physicians, in Indonesia, with a focus on internal medicine specialists—a population critical to health care delivery yet underexplored in low- and middle-income settings (Central Illustration). Methodological rigor was achieved through a mixed-methods approach: self-reported data were triangulated with standardized physical examinations (eg, blood pressure, anthropometry) and laboratory biomarkers (glycemic/lipid profiles), enhancing the validity and objectivity of cardiometabolic risk assessments. The inclusion of 1,064 participants across 39 INASIM branches ensures broad geographical representation, capturing Indonesia’s diverse sociodemographic and occupational landscapes, and enhances generalizability to the nation’s heterogeneous internal medicine specialist workforce. Systematic recruitment via the INASIM branches further mitigated selection bias, ensuring a representative sample of practicing internal medicine specialists.

**Central Illustration:**
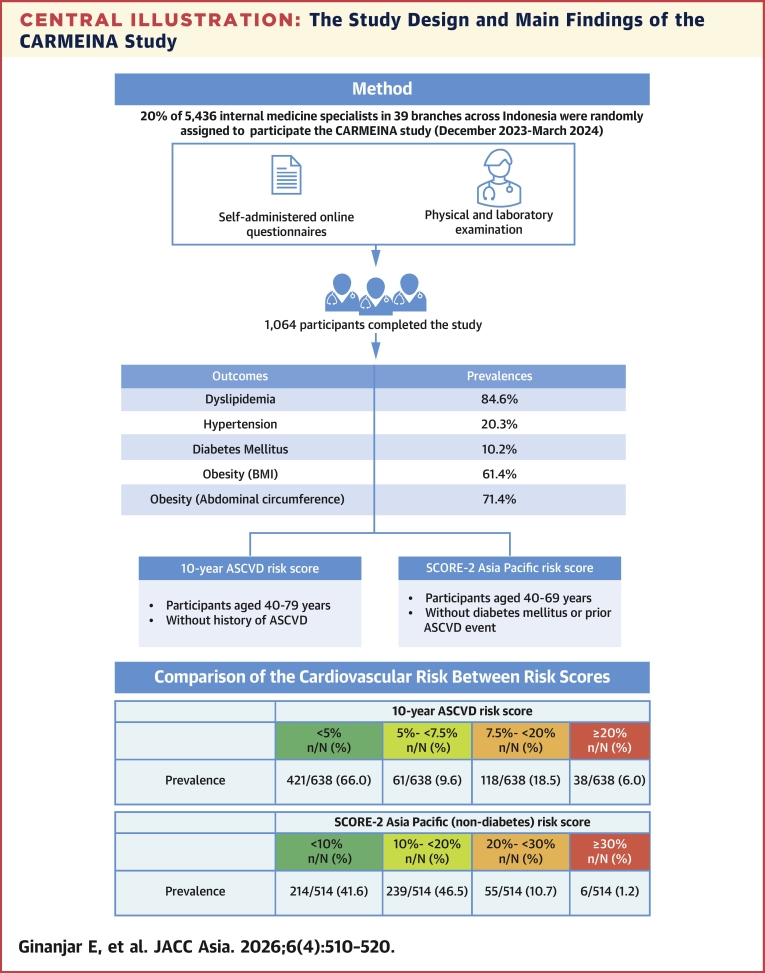
The Study Design and Main Findings of the CARMEINA Study This study aimed to evaluate cardiometabolic risk factors among internal medicine specialists in Indonesia. Participants of the CARMEINA (Cardiometabolic Risk among Internal Medicine Specialists in Indonesia) study were randomly assigned from 39 branches across Indonesia. Data collection occurred in 2 sequential phases, self-administered online questionnaires and physical and laboratory examinations at 39 branch sites. A total 1,064 participants completed the study. Among the participants, the prevalence of dyslipidemia, hypertension, and diabetes mellitus were 84.6%, 20.3%, and 10.2%, respectively. The prevalence of obesity was 61.4% (body mass index) and 71.4% (abdominal circumference). From 638 participants who met the 10-year atherosclerotic cardiovascular disease (ASCVD) risk calculation criteria, most had a risk of <5% (66.0%), whereas of 514 participants who met the SCORE-2 (Systematic Coronary Risk Evaluation-2) Asia Pacific (nondiabetes) risk calculation range, most had a risk of 10% to 20% (46.5%).

### Study limitations

First, the cross-sectional design precludes causal inference between observed health parameters and occupational or lifestyle factors. Second, although self-administered questionnaires are pragmatic for large-scale data collection, they may introduce recall bias, particularly for self-reported lifestyle variables (eg, diet, physical activity). Misclassification bias also could arise from subjective interpretation of questionnaire items, despite standardization. Although internal medicine specialists’ medical expertise may improve self-reporting reliability, the absence of longitudinal validation or external corroboration (eg, electronic health records) limits confirmation of temporal consistency or objective accuracy. Third, 72 participants withdrew because of logistical challenges, including residency in remote areas (limiting access to standardized laboratories), force majeure events (eg, floods), or scheduling conflicts, potentially affecting sample representativeness. Finally, although this study provides foundational insights into internal medicine specialists’ cardiometabolic risks, the absence of direct comparison to the general Indonesian population limits our ability to disentangle occupation-specific risks from broader national health trends. Future studies integrating harmonized national survey data could address this gap.

This study is the first in Indonesia to recruit internal medicine specialists, and it features a quite large sample size compared with other studies that recruit doctors as participants. These findings establish a critical evidence base for occupational health policy in Southeast Asia, where physician well-being remains underprioritized despite its direct impact on health care system resilience. The identification of modifiable risk factors—such as high rates of obesity, dyslipidemia, and sedentary behavior—calls for institutional interventions, including workplace wellness programs and workload redistribution frameworks. Future research should adopt longitudinal designs, incorporate wearable technologies for real-time behavioral monitoring, and employ nested validation sub-studies to address recall and misclassification biases.

## Conclusions

The prevalence of dyslipidemia and obesity was high among internal medicine specialists in Indonesia. The prevalences of highest risk category profile were 6.0% (ASCVD) and 1.2% (SCORE2). Our findings identified the most uncontrolled cardiovascular risk factors among internal medicine specialists in Indonesia. Future intervention to mitigate those risk factors should be considered among internal medicine specialists.

## Funding Support and Author Disclosures

This research did not receive any specific grant from funding agencies in the public, commercial, or not-for-profit sectors. The authors have reported that they have no relationships relevant to the contents of this paper to disclose.
