# Supplementary material for: Cardiometabolic Risk Among Internal Medicine Specialists in Indonesia (CARMEINA): A Nationwide Study
Source: JACC Asia. 2025 Nov 26;6(4):510–20. doi: 10.1016/j.jacasi.2025.09.024 (PMC13080741; doi:10.1016/j.jacasi.2025.09.024)
Supplement: Supplementary Figures 1-4 [file mmc1.docx]

**Supplemental Figure 1**

(A)


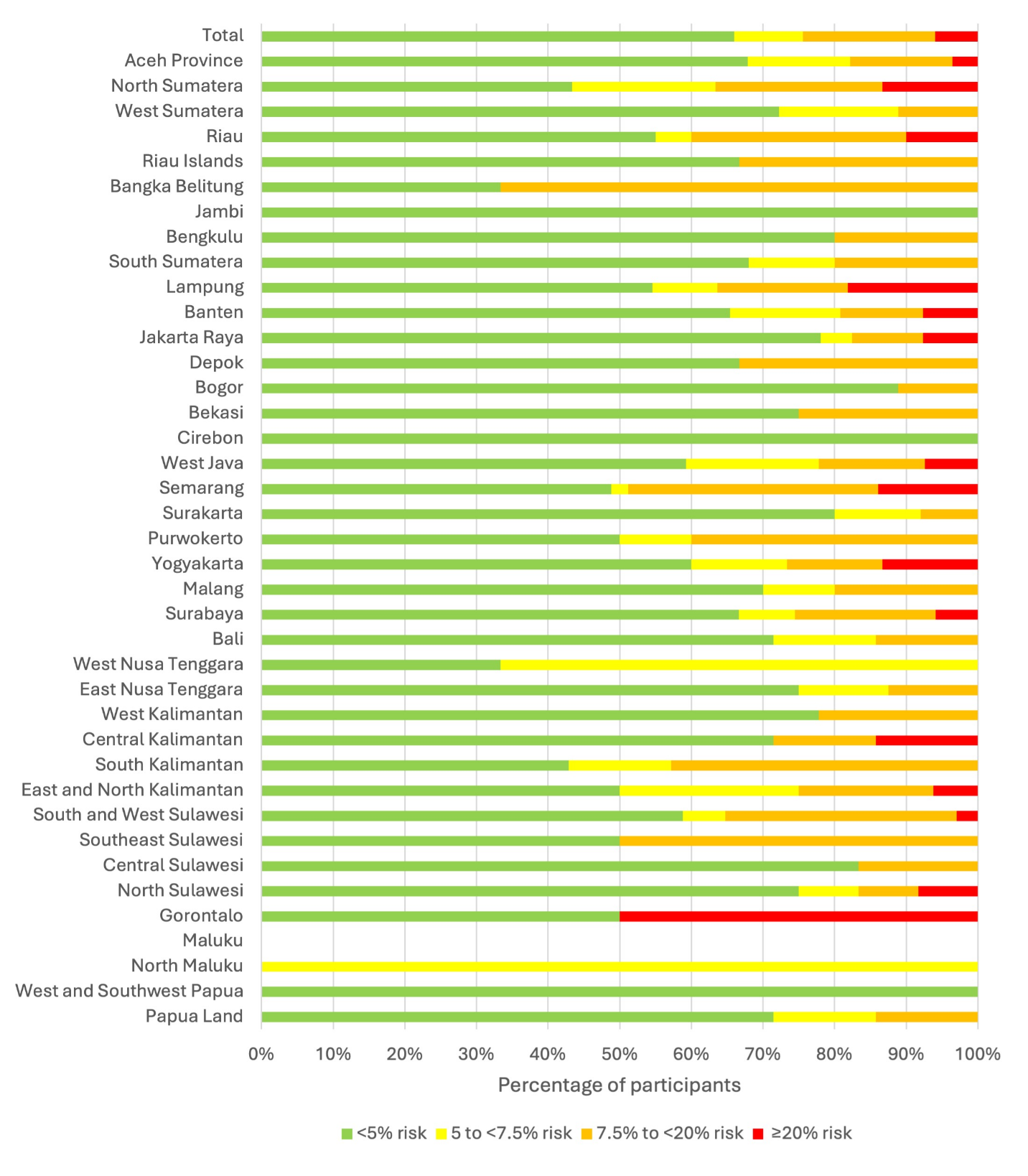


**Figure 1** ASCVD 10-year risk based on (A) location; and gender (B) male, (C) female, cont.


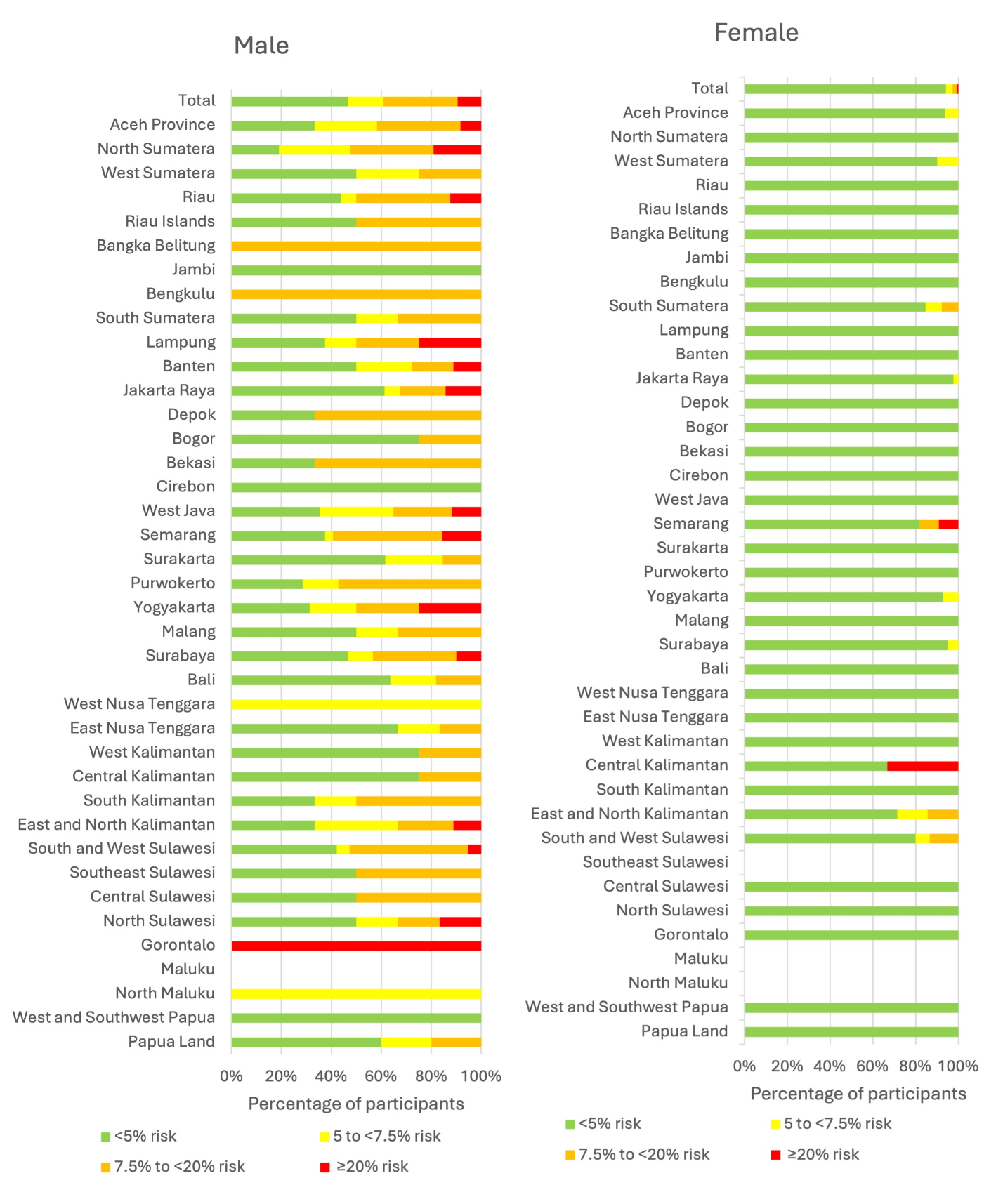


(B)

(C)

**Figure 1** ASCVD 10-year risk based on (a) location; and gender (b) male, (c) female.

**Supplemental Figure 2**

(A)


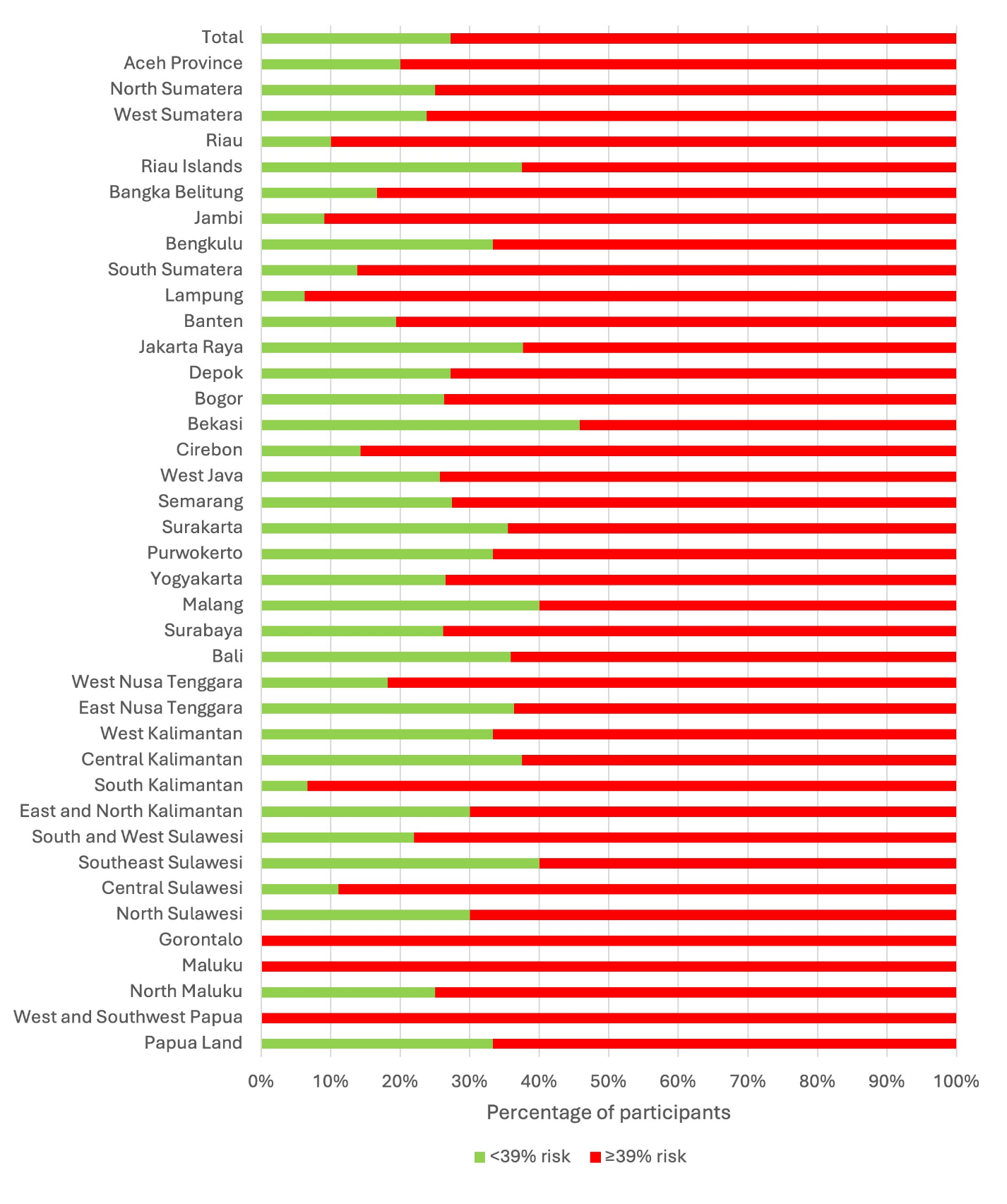


**Figure 2** Lifetime ASCVD risk score based on (A) location; and gender (B) male, (C) female, cont.


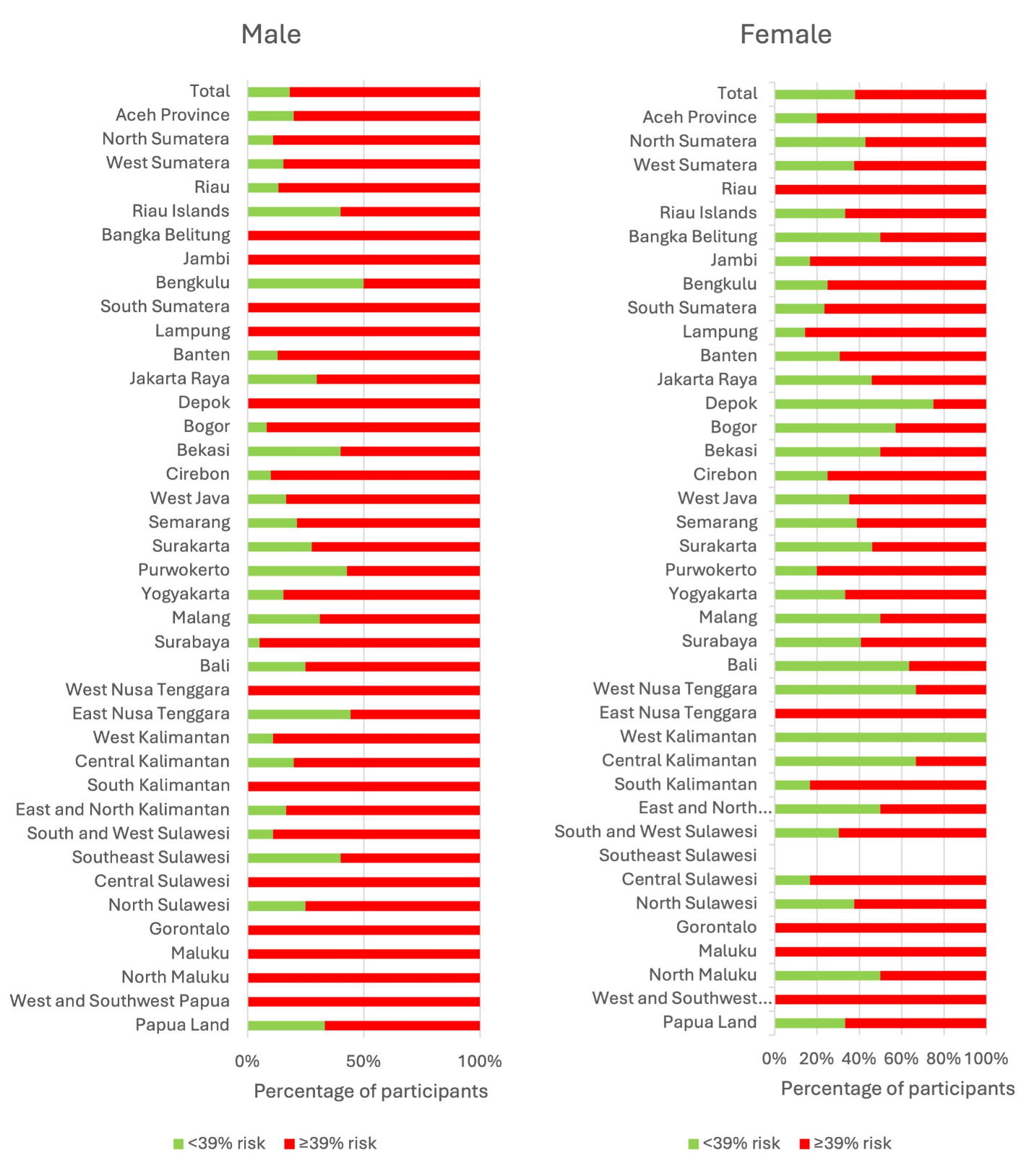


(B)

(C)

**Figure 2** Lifetime ASCVD risk score based on (a) location; and gender (b) male, (c) female.

**Supplemental Figure 3**

(A)


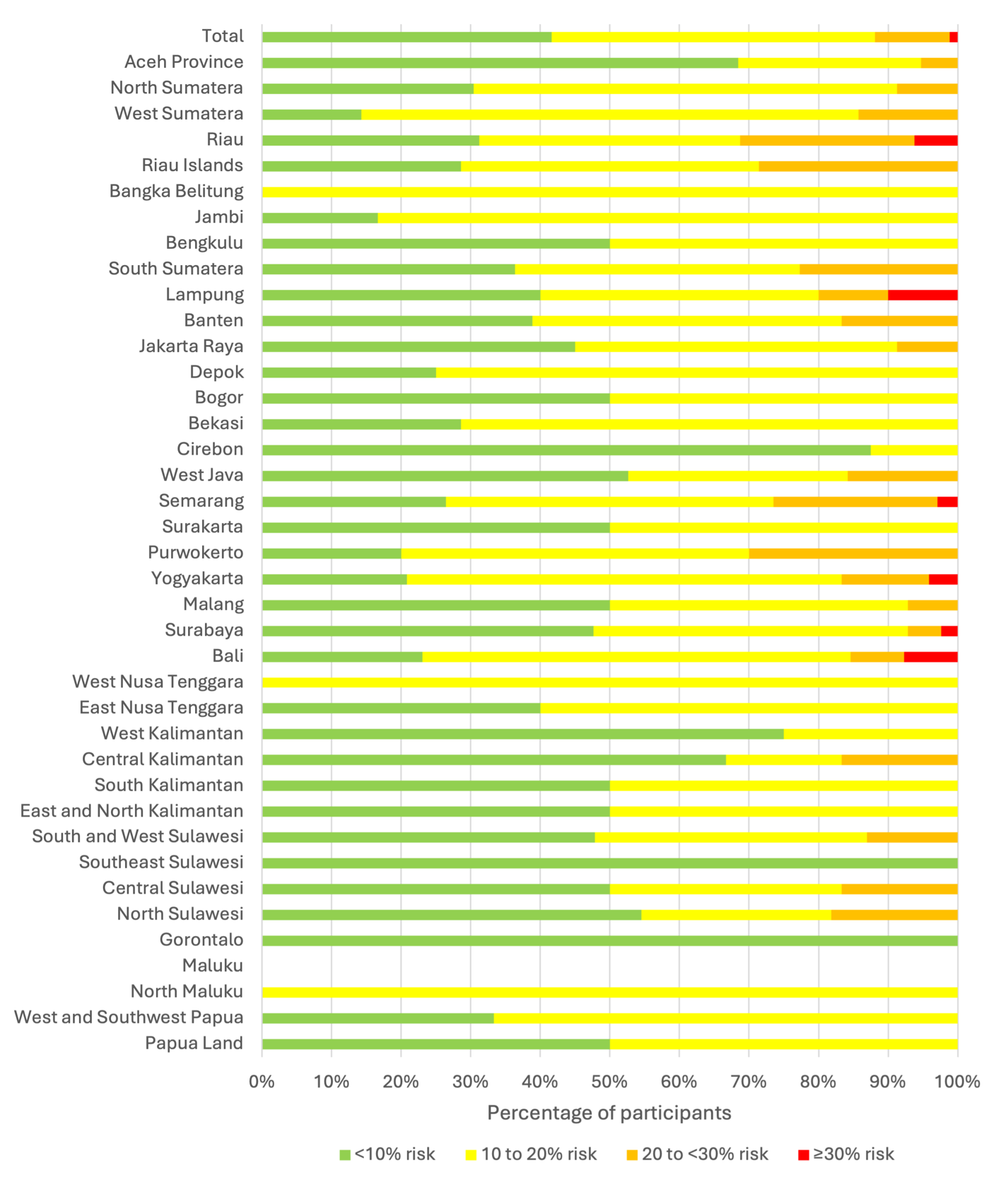


**Figure 3** SCORE-2 Asia Pacific risk score in non-diabetic participants based on (A) location; and gender (B) male; (C) female, cont.


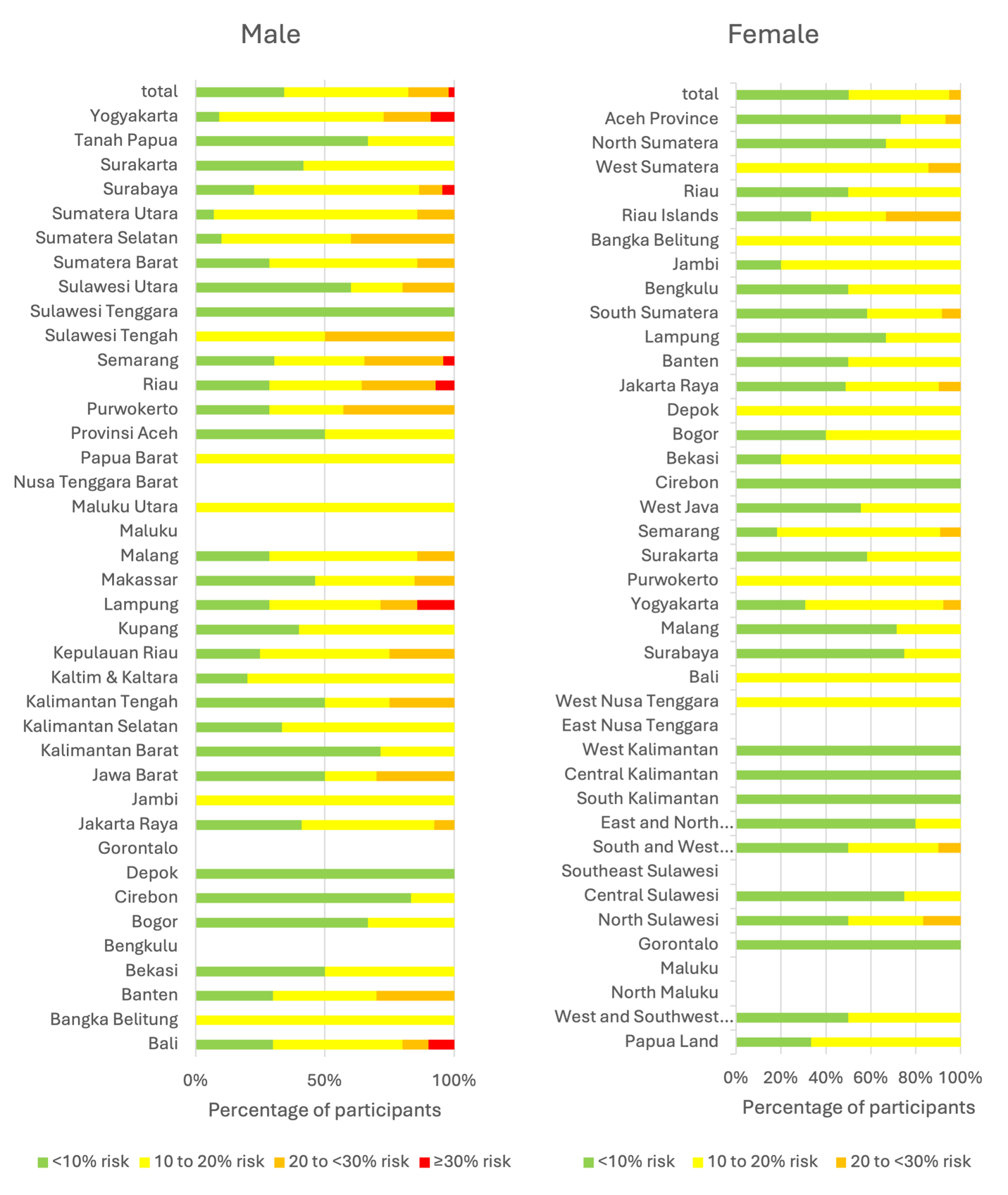


(B)

(C)

**Figure 3** SCORE-2 Asia Pacific risk score in non-diabetic participants based on (a) location; and gender (b) male, (c) female.

**Supplemental Figure 4**


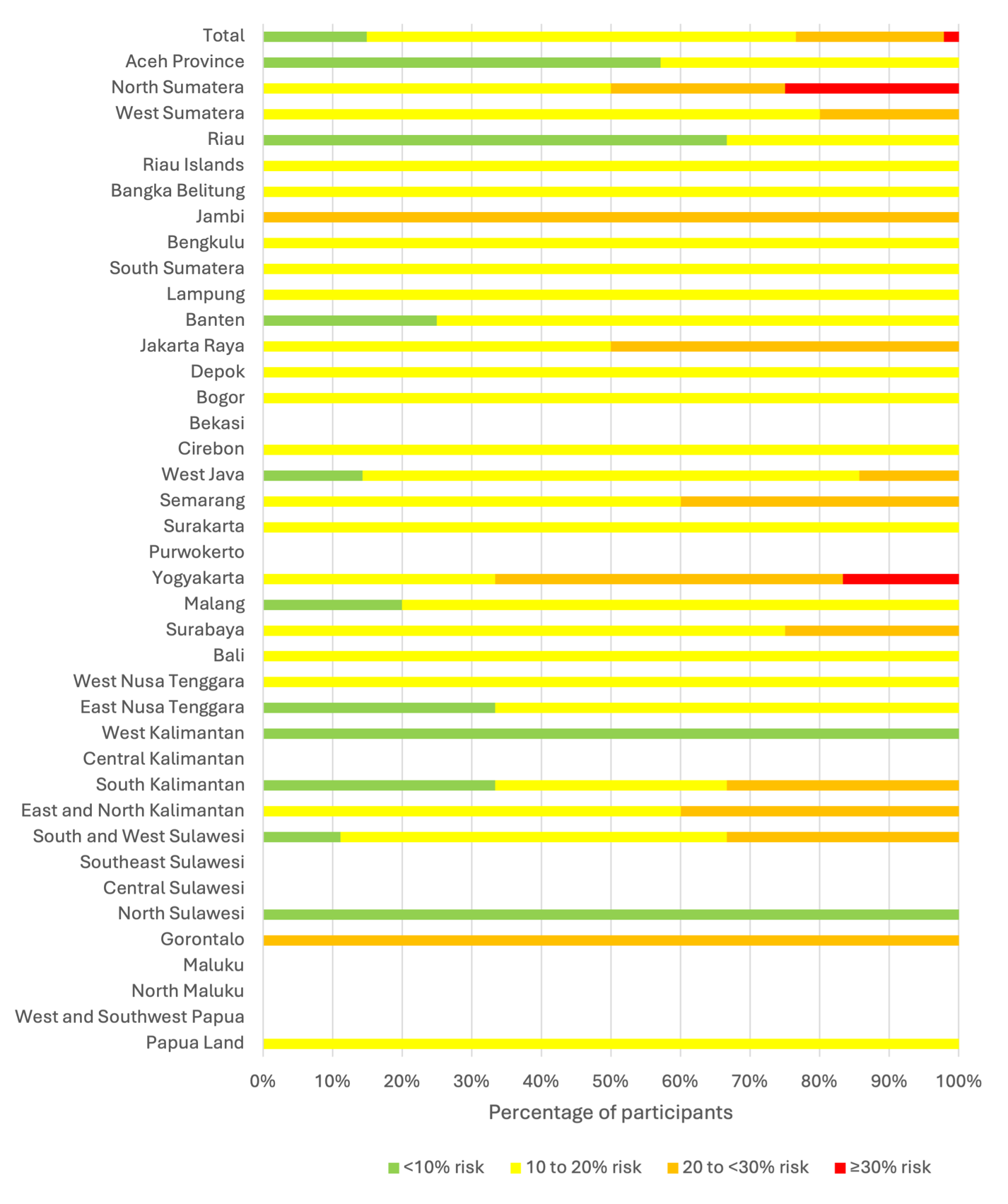


(A)

**Figure 4** SCORE-2 Asia Pacific risk score in diabetic participants based on (A) location; and gender (B) male, (C) female, cont.


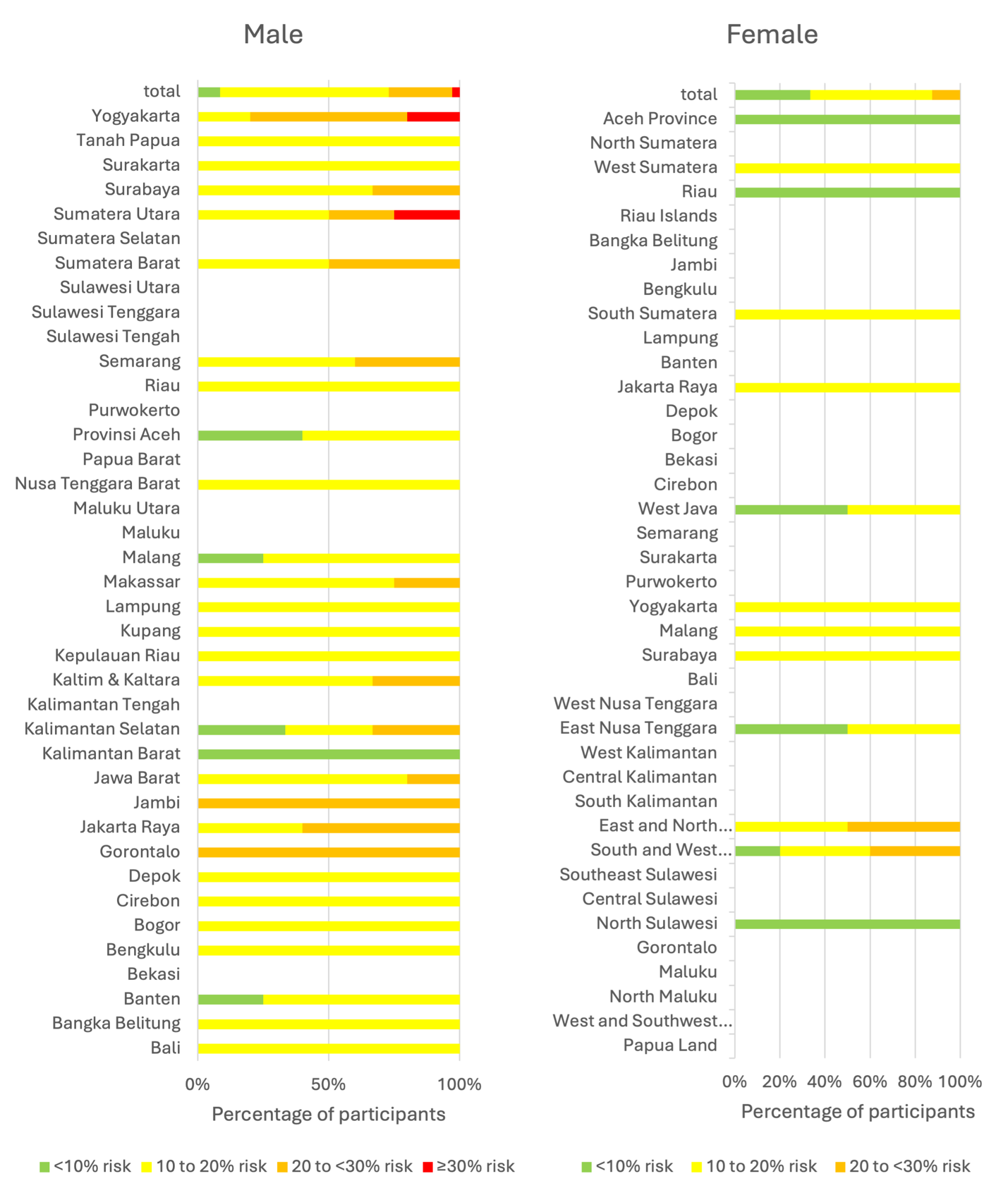


(B)

(C)

**Figure 4** SCORE-2 Asia Pacific risk score in diabetic participants based on (a) location; and gender (b) male, (c) female.
